# Switching CAR T cells on and off: a novel modular platform for retargeting of T cells to AML blasts

**DOI:** 10.1038/bcj.2016.61

**Published:** 2016-08-12

**Authors:** M Cartellieri, A Feldmann, S Koristka, C Arndt, S Loff, A Ehninger, M von Bonin, E P Bejestani, G Ehninger, M P Bachmann

**Affiliations:** 1 University Cancer Center (UCC), Carl Gustav Carus University Hospital TU-Dresden, Tumorimmunology, Dresden, Germany; 2 Cellex Patient Treatment GmbH, Dresden, Germany; 3 Helmholtz-Zentrum Dresden-Rossendorf, Institute of Radiopharmaceutical Cancer Research, Dresden, Germany; 4 GEMoaB Monoclonals GmbH, Dresden, Germany; 5 University Hospital Carl Gustav Carus, Medical Clinic and Policlinic I, Dresden, Germany

## Abstract

The adoptive transfer of CD19-specific chimeric antigen receptor engineered T cells (CAR T cells) resulted in encouraging clinical trials in indolent B-cell malignancies. However, they also show the limitations of this fascinating technology: CAR T cells can lead to even life-threatening off-tumor, on-target side effects if CAR T cells crossreact with healthy tissues. Here, we describe a novel modular universal CAR platform technology termed UniCAR that reduces the risk of on-target side effects by a rapid and reversible control of CAR T-cell reactivity. The UniCAR system consists of two components: (1) a CAR for an inert manipulation of T cells and (2) specific targeting modules (TMs) for redirecting UniCAR T cells in an individualized time- and target-dependent manner. UniCAR T cells can be armed against different tumor targets simply by replacement of the respective TM for (1) targeting more than one antigen simultaneously or subsequently to enhance efficacy and (2) reducing the risk for development of antigen-loss tumor variants under treatment. Here we provide ‘proof of concept' for retargeting of UniCAR T cells to CD33- and/or CD123-positive acute myeloid leukemia blasts *in vitro* and *in vivo*.

## Introduction

Adoptive T-cell therapy with chimeric antigen receptor (CAR) engineered T cells (CAR T cells) has recently shown encouraging clinical results in B-cell malignancies. Approximately 70% of patients showed complete or at least partial response to treatment with autologous T cells equipped with CD19-specific CARs.^1, 2, 3, 4^ CARs are synthetic receptors that retarget genetically engineered T cells against tumor surface antigens. For that purpose, CARs assemble an extracellular binding moiety with an intracellular signaling domain from an activating immune receptor.^5^ The extracellular binding moiety provides the antigen specificity and is commonly a single-chain fragment variable (scFv) derived from a monoclonal antibody (mAb). CARs enable T cells to major histocompatibility complex-independent antigen recognition, and thus major immune escape mechanisms of tumors such as downregulation of major histocompatibility complex molecules are efficiently bypassed.^6^ Despite the clinical success of CD19-specific CAR T cells, there are still a number of conceptual limitations inherent to this treatment strategy. Currently used conventional CARs have one target specifity. Such a monospecific targeting approach harbours the risk for the development of tumor escape variants.^7, 8^ More importantly, the fixed antigen-binding moiety on CAR-modified T cells provides no means of direct control over ongoing CAR T-cell reactivity. After infusion, CAR T cells expand in response to their antigen by 100- to 10 000-fold, making the magnitude of their reactivity unpredictable. Adverse reactions, due to either inappropriate on-target, off-tumor reactions against healthy tissue or excessive on-target, on-tumor reactions against heavy tumor loads, are thus difficult to handle and pose a high risk during treatment. In fact, approximately one-third of patients treated in recent trials experienced severe fevers and inflammations and all patients suffer from ongoing B-cell aplasia, as long as CD19-specific CAR T cells are present in their circulation. Lack of B cells is manageable clinically by intravenous immunoglobulin administration; still, patients have a high risk for opportunistic infections. In other instances such an on-target, off-tumor effect may be not acceptable or even life-threatening.^9, 10^ Thus, without an additional safety switch CAR T cells can only be redirected against truly tumor-restricted antigens, which are very rare, or antigens with highly restricted tissue expression, as it is the case for CD19. This limits the application of CAR treatment to very few tumor entities.

For instance, acute myeloid leukemia (AML) is a heterogeneous leukemic disease with still unmet medical need for the development of new treatment modalities, as 50–70% of patients experience a relapse after they initially respond well to standard induction chemotherapy. A recent in-depth analysis on >300 patient samples revealed that CD33 and CD123 are expressed either in combination or alone on nearly 100% of all AML blasts.^11^ Therefore, both antigens seem to be ideal targets for immunotherapy.^12, 13^ However, redirecting T cells against CD33 or CD123 with single-specific CARs for AML treatment is hampered by the fact that both antigens are expressed not only on AML blasts, but are also present on hematopoietic stem cells, on progenitor and mature hematopoietic cells of the myeloid lineage and on endothelial cells.^14^ To extend the application range of CAR T cells, we developed a flexible modular CAR platform (UniCAR) that allows switching CAR T cells on and off in a controlled manner. Here we provide ‘proof of function' of the UniCAR concept by successfully retargeting human UniCAR engineered T cells against the AML antigens CD33 and CD123.

## Materials and methods

### Cell lines

Chinese Hamster ovarian (CHO) cells, MOLM-13 and MV4-11 were cultured in complete RPMI-1640 medium and OCI-AML3 in complete Dulbecco's modified Eagle's medium.^15^ Cells were maintained at 37 °C in a humidified atmosphere of 5% CO_2_.

### Generation of UniCAR vectors

The UniCAR-binding domain is based on the mAb anti-La 5B9 that recognizes a continous sequence of 10 amino acids (5B9 tag) of the nuclear protein La/SS-B.^16^ The cloning of the humanized anti-La 5B9 scFv and generation of the hinge, transmembrane and signaling domain of the CAR was recently described in detail.^16, 17^ In order to facilitate the fluorescence-activated cell sorting (FACS) analysis of UniCAR expression on the surface of transduced T cells, we fused another peptide epitope of 18 amino acids (E-tag18) and an additional linker consisting of four glycines followed by one serine (G_4_S_1_) between the UniCAR scFv and the CD28 coding region. The E-tag can be detected by another anti-La mAb (7B6) that was generated by standard hybridoma fusion technique and identified to be reactive against the introduced epitope sequence.^18^ The CAR signaling and stop constructs were subsequently cloned into lentiviral vector backbone p6NST60.^19^ The open reading frames of the CAR constructs were fused to an enhanced green fluorescent protein open reading frames separated by a 2pA protease site derived from the *Thosea asigna* virus that allows an independent translation of CAR and enhanced green fluorescent protein from a single mRNA in modified T cells.^20^

### Construction and expression of recombinant antibodies

Cloning of the targeting module directed against CD33 has been described elsewhere.^15, 21^ The targeting module (TM) directed to CD123 is based on the mAb 7G3.^22^ The sequences encoding its variable light and heavy chain were connected by a three times repeat of G_4_S_1_ and fused to the 5B9 tag followed by a his tag. For the bispecific TM the two scFvs were connected by a linker comprising the amino acid sequence of the 5B9 epitope. Stable recombinant TMs producing CHO cell lines were established by lentiviral gene transfer and recombinant proteins were purified from cell supernatants via Ni-NTA affinity chromatography followed by analysis of protein concentration and purity through SDS–polyacrylamide gel electrophoresis and immunoblotting as previously described.^23^

### Isolation and lentiviral transduction of human T cells

Isolation of primary human T cells from peripheral blood mononucleated cells, transduction procedure and maintenance of T cells was performed as recently described.^17^ Genetically modified T cells were purified by cell sorting using a FACSAria II (BD Biosciences, Heidelberg, Germany). After purification, T cells were rested in RPMI supplemented with cytokines for additional 5–6 days. Media were substituted for complete RPMI lacking any recombinant cytokines 24 h before experiments were performed.

### Flow cytometry analysis

Isolated T cells were stained with fluorochrome-labeled mAbs directed against human CD4/VioBlue (clone VIT4, Miltenyi Biotec, Bergisch Gladbach, Germany), CD3/PE-Cy7 (clone UCHT1, BioLegend, Uithoorn, The Netherlands), CD8/APC (clone RPA-T8, BD Biosciences) and CD25/PE (clone 4E3, Miltenyi Biotec). For detection of CAR surface expression T cells were incubated with mAb anti-La 7B6 and subsequently stained with phycoerythrin-labeled goat anti-mouse IgG (Beckmann Coulter, Krefeld, Germany).^17^ Samples were analyzed using the MACSQuant Analyzer and the MACSQuantify software (Miltenyi Biotec).

### Cytotoxicity assay

For analysis of their cytotoxic potential, modified T cells were cultured with antigen-positive tumor cells in the presence or absence of TMs at the indicated concentrations. The specific target cell lysis at indicated time points was determined by standard chromium release assays or flow cytometry-based viability assays using the MACSQuant Analyzer as recently described.^24^ For flow cytometry-based viability assays target cells were labeled with live cell-dye eFluor 670 (eBioscience, Frankfurt, Germany) to distinguish them from effector cells.

### T-cell expansion assay

In order to assess expansion rates of CAR-armed T cells, absolute T-cell numbers were quantified in flow cytometry-based viability assays using a MACSQuant Analyzer and MACSQuantify software as described elsewhere.^17, 24^

### Cytokine-release assay

Cell-free supernatants were harvested after 24 h from cultures to determine cytokine concentrations by using the OptEIA Human IFN-γ, OptEIA Human IL-2 and OptEIA human tumor necrosis factor enzyme-linked immunosorbent assay (ELISA) Kits (BD Biosciences) or a ProcartaPlex Multiplex Human Th1/Th2 cytokine panel (eBioscience).

### *In vivo* experiments in NOD/SCID IL2Rγ^−/−^ (NSG) mice

#### *In vivo* toxicity studies

Genetically modified human T cells were intravenously (i.v.) injected into NSG mice at the indicated concentrations. Mice were carefully examined on a daily basis for signs of illness and their body weight was monitored in weekly intervals.

#### *In vivo* pharmacokinetic studies

NSG mice were injected either i.v. or intraperitoneally (i.p.) with 250 ng per g body weight of the dual-specific TM CD123-CD33 and blood samples were taken at the indicated time periods after injection. TM concentrations in peripheral blood samples were determined with an in-house ELISA. Briefly, 96-half-well plates were coated with anti-La 5B9 mAb (5 μg/ml) and incubated with diluted blood samples. For detection of bound TM a horseradish peroxidase-conjugated anti-HIS mAb (DAKO, Eching, Germany) was used. A standard curve with diluted purified TM was established to estimate TM concentrations in blood samples.

#### NOD/SCID IL2Rγ^−/−^ mouse AML bone marrow xenograft model

The 8–10-week-old NSG mice were injected i.v. with 1 × 10^6^ genetically modified human T cells. On day 28 after T-cell injection, 5 × 10^5^ MOLM-13 cells were given i.v. to the mice and treatment was started 5 days later. For this purpose, 250 ng per g mouse body weight of the TM was injected i.p. twice a day over 2 consecutive days. Mice were killed when severe derogation of health occurred because of tumor development and single-cell suspensions from bone marrow obtained from femur and tibia of the left hind leg were prepared. Erythrocytes were removed by lysis and nucleated cells were stained with anti-mouse CD45.1/PE-Cy7 (clone A20, eBioscience), anti-human CD3/APC-eFluor 780 (clone SK7, eBioscience), CD19/APC (clone HIB19, BD Biosciences), CD33/PE (clone HIM3-4, eBioscience) and CD45/AlexaFluor 700 (clone HI30, BioLegend) mAbs. Doublet discrimination was routinely carried out and dead cells were excluded by 4',6-diamidino-2-phenylindole (DAPI) staining (Sigma-Aldrich, Steinheim, Germany). All measurements were performed on a BD LSRII FACS machine (BD Biosciences). Data analysis was realized using FlowJo software (Tree Star Inc., Ashland, OR, USA).

### Statistical analysis

Statistical analysis was performed with GrapPad Prism software version 5.0 (GraphPad Software Inc., San Diego, CA, USA).

### Study approval

Blood sampling from healthy individuals and patients was approved by the local ethics committee of the university hospital of the medical faculty of Carl-Gustav-Carus TU-Dresden (EK27022006). All animal experiments were performed according to the German animal protection law with permission from the responsible local authorities and ethics committee (Sächsische Landesdirektion, 24-9168.11-1/2013-32). Mice were kept under standardized environmental conditions and received autoclaved food, water and bedding.

## Results

### UniCAR engineered T cells eliminate AML cells with the help of individual CD33- and CD123-specific targeting modules

**Figure 1:**
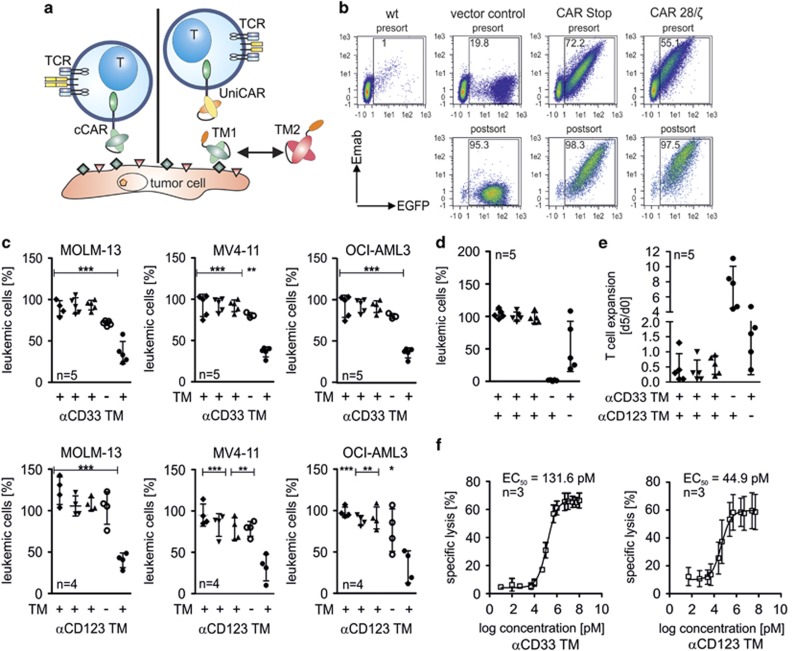
UniCAR T cells eradicate AML cells upon redirection with CD33-specific (αCD33 TM) or CD123-specific (αCD123 TM) targeting modules. (**a**) Schematic representation of T cells engineered with a conventional CAR (left panel, cCAR) or a UniCAR (right panel). For explanation, see text. (**b**) UniCAR surface expression was detected as described in the Materials and methods section. For all experiments, transduced T cells were sorted to >90% purity to allow comparison between different human donors. (**c**) Reduction of leukemic cells from three AML cell lines after 24 h of incubation with 2 × 10^4^ engineered human T cells isolated from healthy donors in the presence (+) or absence (−) of 0.1 nM TM in an effector-to-target (e/t) ratio of 1:1. Samples were normalized to a target cell control without any T cells. Engineered T cells expressed either UniCARs containing a dual CD28/CD3-ζ signaling domain (open and closed circles), UniCARs lacking any signaling domain (head up triangle), enhanced green fluorescent protein (EGFP) marker protein (head down triangle) or where not genetically modified (rhombus). (**d**, **e**) Experimental set-up was similar to (**c**) but a lower e/t ratio of 1:5 was chosen. (**d**) The number of living MOLM-13 target cells was normalized to a control sample without any T cells. (**e**) T-cell expansion was calculated as the ratio of T cells present in the samples after 120 h (d5) to the number of cells seeded at the start of the experiment (d0). (**f**) Effective TM concentration required for lysis of MOLM-13 AML cells was determined after 24 h of cultivation with UniCAR T cells. TMs were added at the indicated concentrations. Statistical analysis for (**c**) was performed using nonparametric one-way analysis of variance (ANOVA; Kruskal–Wallis test) and *post hoc* Dunn's multiple comparison test. Results are indicated for UniCAR modified T cells plus TMs versus other samples (*P<0.05, **P<0.01, ***P<0.001).

As summarized in the Introduction section, the UniCAR technology splits signaling and antigen-binding domains of conventional CARs into two individual components (Figure 1a). The cellular component is a CAR, structurally similar to conventional second-generation CARs with a combined CD28/CD3-ζ intracellular signaling domain (Supplementary Figure 1a). As in conventional CAR constructs, the extracellular domain consists of an scFv binding domain. In contrast to conventional CARs, the scFv in universal CARs does not recognize a cell surface antigen but instead a short nonimmunogenic peptide motif of 10 amino acids (5B9 tag) derived from the human nuclear autoantigen La/SS-B.^16^ Thus, T cells engineered to express UniCARs remain inactive after reinfusion, as this UniCAR target is not available on the surface of intact cells under physiological conditions.^25^ The ultimate antigen specificity of the system is provided separately by a second component, a TM comprising a binding domain directed against a tumor antigen (for example, a tumor-specific scFv) fused to the 5B9 tag recognized by the UniCAR scFv (Figure 1a). Obviously, TMs are interchangeable, thus, adding a high flexibility to the system.

Human primary T cells were genetically modified by lentiviral gene transfer and surface expression of UniCARs demonstrated (Figure 1b). For all experiments, modified human UniCAR T cells were purified by flow cytometric cell sorting beforehand to ensure that results are independent of initial transduction efficacy and comparable between donors (Figure 1b). First, two individual TMs were generated either recognizing CD33 or CD123. Three different AML lines with varying CD33 and CD123 surface expression were used as target cells in cytotoxicity assays to explore the dependence of UniCAR responses in antigen density. For CD33, antigen density ranged from a few thousand molecules per cell on OCI-AML3 cells to approximately half a million on MOLM-13.^15^ No correlation was observed between target antigen densities and killing efficacy by TM-redirected UniCAR T cells (Figure 1c). UniCAR modified T cells were even able to efficiently lyse AML cells at low effector-to-target ratios of 1:5 over prolonged time periods if sufficient TM was supplied (Figure 1d). The TM-mediated engagement of target cells stimulated proliferation of UniCAR T cells, leading to a net expansion over time (Figure 1e). Titration experiments revealed that low pM TM concentrations already induce efficient MOLM-13 lysis (Figure 1f). Taken together, these experiments show that TMs tagged with the 5B9 tag recognized by UniCAR modified T cells are both necessary and sufficient to redirect UniCAR T cells against antigen-positive target cells.

### Simultaneous dual targeting enhances leukemic cell lysis by UniCAR modified T cells

**Figure 2:**
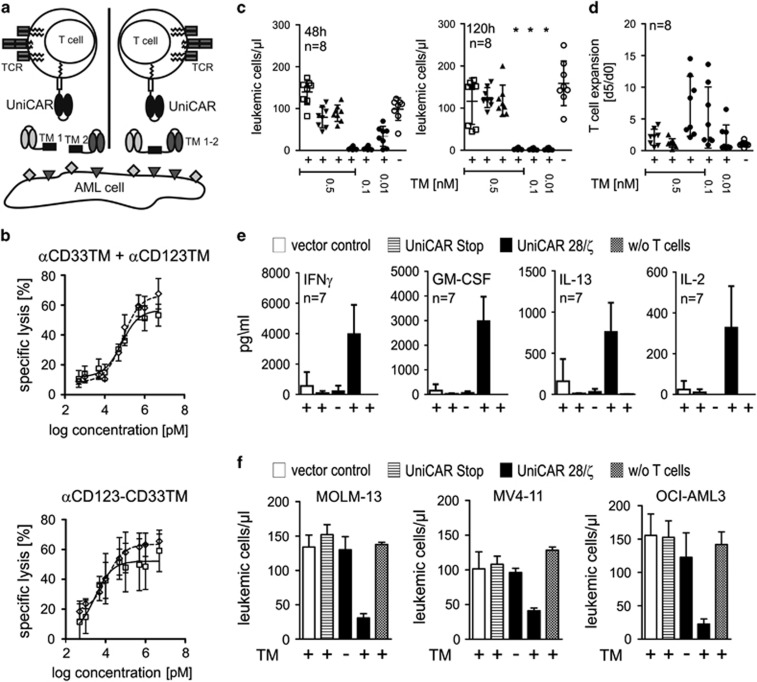
Dual retargeting of UniCAR modified T cells against CD33 and CD123 enhances leukemic cell lysis. (**a**) Because of its modular nature, the UniCAR technology allows a redirection of UniCAR modified T cells against two antigens simultaneously or consecutively using either two monospecific TMs (TM 1+TM 2, left side) or combined dual-specific TMs (TM1-2, right side). (**b**) Concentration–response curves for combined CD33- and CD123-specific retargeting of UniCAR T cells. UniCAR T cells were incubated in an effector-to-target (e/t) cell ratio of 1:1 with ^51^Cr-labeled MOLM-13 (*n*=4, open squares) and OCI-AML3 (*n*=2, open rhombus) for 24 h. EC_50_ values were determined for anti-CD33 (αCD33 TM)+anti-CD123 TM (αCD123 TM): EC_50_ MOLM-13=70.2 pM, EC_50_ OCI-AML3=80.2 pM, for anti-CD123-anti-CD33 TM (αCD123-CD33 TM): EC_50_ MOLM-13=2.9 pM, EC_50_ OCI-AML3=11.7 pM. (**c**, **d**) Human engineered T cells from healthy donors or AML patients were incubated with 5 × 10^4^ CD3^-^CD19^-^ leukemic cells from AML patients in the presence (+) or absence (−) of anti-CD123-CD33 TM at the indicated concentrations and an e/t ratio of 1:1. T cells expressed either UniCARs containing a dual CD28/CD3-ζ signaling domain (open and closed circles), UniCARs lacking any signaling domain (head up triangle) or enhanced green fluorescent protein (EGFP) marker protein (head down triangle). (**c**) The number of living target cells after 48 h (left panel) and 120 h (right panel) is shown and compared with control AML samples without adding T cells (open squares). (**d**) T-cell expansion was calculated as the ratio of T cells present in the samples after 120 h (d5) to the number of cells seeded at the start of the experiment (d0). (**e**) T cell-specific cytokine secretion was determined from supernatants taken after 48 h from the experiments shown in (**c**). (**f**) Engineered T cells from AML patients were incubated with 2 × 10^4^ AML target cells in the presence (+) or absence (−) of 0.5 nM TMs in an e/t ratio of 1:1. Number of living target cells was determined by flow cytometry after 24 h and compared with a control sample with target cells and TM but without T cells. Results from one representative donor are shown. Statistical analysis for (**c**) was performed using nonparametric one-way analysis of variance (ANOVA; Kruskal–Wallis test) and *post hoc* Dunn's multiple comparison test. Results are indicated for UniCAR modified T cells plus TMs versus other samples (*P<0.05).

The modular nature of the UniCAR technology prompted us to consider the possibility of simultaneously redirecting UniCAR modified T cells against multiple target antigens either by using more than one individual monospecific TM or by generating a dual-specific TM (Figure 2a). For this purpose, a dual-specific TM against CD33 and CD123 was constructed and titrated in a cytotoxicity assay on MOLM-13 and OCI-AML3 cells (Figure 2b). Interestingly, UniCAR T cells retargeted by the dual-specific TM lysed AML cell lines more efficiently than simultaneous application of the two monospecific TMs at equal molar ratios, as indicated by the lower half-maximal effective TM concentration (EC_50_) values required to induce efficient AML cell lysis (Figure 2b). Hence, competition of two independent TMs for UniCAR-binding sites may attenuate killing response compared with a dual-specific TM that consists of two antigen-binding arms but only one target epitope to interact with the UniCAR scFv (Figure 2a). Nonetheless, the combination of monospecific anti-CD33 with anti-CD123 TMs as well as the single dual-specific anti-CD123-CD33 TM were able to efficiently redirect UniCAR modified T cells of healthy donors against all tested AML cell lines in an effector-to-target ratio of 1:5 and significantly reduce blast numbers (Supplementary Figure 1b). As seen before, engagement of UniCAR modified T cells by specific TMs leads to proliferation and net expansion of T cells over time (Supplementary Figure 1c). Next, we evaluated the ability of the dual-specific TM to redirect UniCAR modified T cells against primary AML samples. T and B cell-depleted leukocytes from patients with AML, consisting of ⩾80% blasts (analyses by flow cytometry), were incubated in a fixed ratio of 1:1 with third-party UniCAR T cells from either healthy donors or AML patients. As shown in Figure 2c, T cells modified with signaling UniCARs eradicated AML blasts in the presence of the dual-specific TM within 48 h. After a prolonged incubation, UniCAR T cells mediated efficient AML blast lysis even at low TM concentrations equivalent to EC_50_ values determined on AML cell lines (Figure 2c). In contrast, even after a prolonged co-incubation time, alloreactivity of T cells against allogeneic AML cells was negligible, although samples were not human leukocyte antigen matched (Figure 2c). Corresponding flow cytometry plots for one representative experiment are shown in Supplementary Figure 1d. These analyses also demonstrate the strict dependence of UniCAR T-cell activation on the presence of antigen-specific TMs, indicated by upregulation of the T cell-specific activation marker CD25 (Supplementary Figure 1d). Interestingly, expansion of TM-redirected UniCAR T cells correlated directly to TM concentrations in these experiments (Figure 2d). Supernatants harvested after 48 h from these experiments were analyzed for the presence of T cell-specific cytokines. As expected, an increased amount of cytokines was only detected in those samples containing UniCAR engineered T cells and the dual antigen-specific TM in the presence of AML blasts (Figure 2e). Most abundant in the samples were interferon-γ, granulocyte macrophage colony-stimulating factor and interleukin (IL)-13 (Figure 2e). In addition, TM-activated UniCAR T cells also secreted IL-2 (Figure 2e), IL-12p70, IL-4, IL-18 and tumor necrosis factor-α (Supplementary Figure 1e), but in smaller amounts compared with the above-mentioned cytokines. The ability of UniCAR engineered T cells derived from AML patients to lyse AML cell lines was tested in *in vitro* cytotoxicity assays (Figure 2f). Notably, killing efficacy of patient-derived UniCAR T cells upon antigen-specific redirection with the dual-specific anti-CD123-CD33 TM was comparable between T cells from healthy donors and patients with AML (Figure 2f).

### *In vivo* dual (re)targeting delays leukemia outgrowth

**Figure 3:**
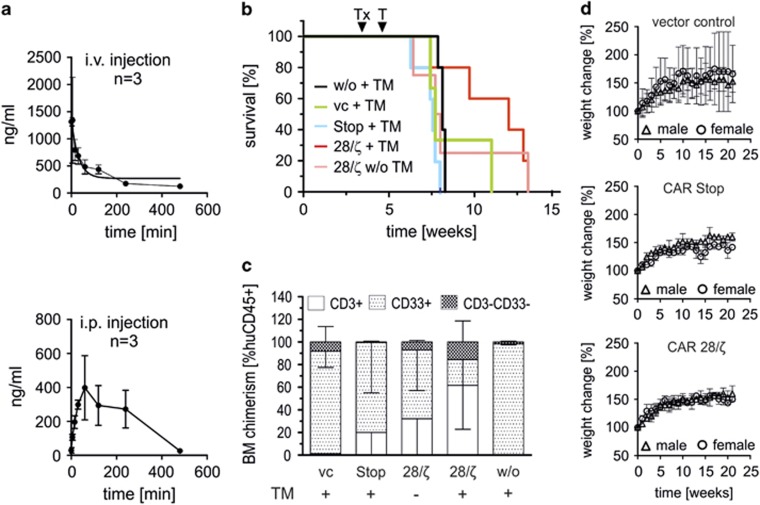
UniCAR engineered T cells delay AML engraftment in a TM-dependent manner *in vivo*. (**a**) Pharmacokinetics of the dual-specific anti-CD123/CD33 TM (αCD123-CD33 TM) in peripheral blood of NSG mice upon i.v. injection via the tail vain or i.p. injection. Concentration of TM in blood samples was determined by ELISA. (**b**, **c**) Short-term treatment with anti-CD123-CD33 TM enhances the survival of NSG mice in an aggressive AML model. 1 × 10^6^ human T cells engineered to express functional UniCARs (28/ζ+TM, red line, *n*=5), UniCARs lacking any signaling domain (Stop+TM, blue line, *n*=5) or expressing only enhanced green fluorescent protein (EGFP) marker protein (vc+TM, green line, *n*=3) were i.v. injected into NSG mice. After 28 days, 5 × 10^5^ MOLM-13 were transferred into NSG mice via i.v. injection (Tx) and treatment with anti-CD123-CD33 TM (T) was started 5 days later. For this purpose, 250 ng TM per g mouse body weight was injected i.p. twice a day over 2 consecutive days. As additional controls, one group of mice was transplanted only with MOLM-13 and treated with anti-CD123-CD33 TM (w/o+TM, black line, *n*=4), and another group of mice was transplanted with functional UniCAR T cells and MOLM-13, but not treated with anti-CD123-CD33 TM (28/ζ w/o TM, light red line, *n*=4). (**b**) Survival curves of experimental groups and (**c**) percentage of CD3^+^ T cells, CD33^+^ MOLM-13 cells and human CD45^+^CD3^-^CD33^-^ cells in the bone marrow of killed mice are shown. (**d**) To evaluate *in vivo* toxicity of UniCAR modified T cells, 1 × 10^6^ engineered human T cells were i.v. injected into NSG mice. Body weight of mice was monitored in weekly intervals and expressed as percentage body weight change over time.

Next, we wanted to show that the UniCAR system is also working *in vivo.* After i.v. bolus injection, the dual-specific anti-CD123-CD33 TM showed a rapid clearance from peripheral blood of NSG mice with a half-life of ∼1 h (Figure 3a, upper panel). The i.p. injection extended the half-life, but lowered the maximal concentration versus i.v. injection (Figure 3a, lower panel). Nevertheless, at a dosage as low as 250 ng TM per g body weight, the plasma concentration still exceeded the *in vitro* EC_50_ value for at least 6–8 h (Figure 3a). Thus, a dosage of 250 ng/g per i.p. injection for 2 days was chosen for an *in vivo* treatment experiment. Despite the shortness of the therapeutic intervention, treatment delayed the occurrence of lethal AML (Figure 3b) and led to a shift in the composition of engrafted human leukocytes from CD33^+^ AML blasts to CD3^+^ T cells in the bone marrow (Figure 3c).

### UniCAR T cells remain inert *in vivo* and show no signs of toxicity

TM-independent on-target, off-site activation of UniCAR modified T cells by recognition of the endogenous La peptide motif would be a major concern for clinical application of the UniCAR platform. As this peptide motif is highly conserved between humans and mice, the parental La peptide-recognizing mAb crossreacts with the human and murine La protein.^16^ Thus, mouse models engrafted with human UniCAR T cells can be used for preclinical toxicology studies. Transplantation of unmodified human T cells into NSG mice leads to the induction of xenogeneic graft-versus-host disease.^26^ In case of the recognition of mouse La antigen by UniCARs, one would expect that an activation of UniCAR engineered T cells finally results in accelerated xenogeneic graft-versus-host disease effects. However, we observed no signs of severe UniCAR-driven xenogeneic graft-versus-host disease effects in this model; for example, there were no differences in weight development between UniCAR T cell-engrafted mice or controls (Figure 3d). Similarly, no signs of other kinds of toxicities were detected (data not shown). Consequently, UniCARs did not cause off-tumor, on-target side effects in NSG mice, although the target epitope 5B9 is present in mouse La protein.

## Discussion

CD33 and CD123 are promising targets for immunotherapy of AML. Recent analysis by our group revealed that CD33 and CD123 are present on 87% and 78% of AML samples, respectively.^11^ However, ∼26% of AMLs are negative for one of both antigens and represent a significant therapeutic gap for any monotherapeutic approach.^11^ Feasibility of immunotherapy against CD33 was proven both preclinically and clinically^15, 21, 27, 28^ and hematopoietic stem cells seem not to be affected,^21, 29^ although the presence of CD33 on hematopoietic stem cells is still under debate.^30^ Preclinical studies with CD33 single-specific CAR-armed T cells already demonstrated antileukemic efficacy both *in vitro* and *in vivo*.^29, 31^ However, it is of great concern that CD33-specific effector immune cells will abrogate myeloid hematopoiesis as long as they are present in the circulation, as CD33 is expressed on myeloid progenitors and mature cells. Similar to CD33, CD123 expression is also reported from progenitor and mature hematopoietic cells of the myeloid lineage, whereas it is absent on uncommitted hematopoietic stem cells.^14, 32^ CD123 expression is also detected on leukemic stem cells, making it an ideal target for an immune attack harming not only the majority of mature blasts, but also the cancer stem cell reservoir.^33, 34^ Antibody-based targeting of CD123 has been reported to be well tolerated,^35^ but the more powerful approach of targeting CD123^+^ AML cells with CAR T cells markedly impairs human myeloid hematopoiesis in xenograft mouse models.^36^ This effect might be less pronounced in *in vitro* toxicity assays.^22, 37^ Nevertheless, as CAR T cells are permanently active as long as the antigen is present, clinical application of CD33− or CD123 CAR T cells bears the risk of an ongoing impairment of human hematopoiesis as long as the cells are present in a patient.^38^

Our platform technology extends the classical CAR T-cell approach by splitting conventional CARs into two components, a physiologically silent CAR and a soluble TM determining the tumor specificity. Both components can form a complex working like a conventional CAR. Our results demonstrate that activation and induction of effector functions of such UniCAR engineered T cells is strictly dependent on the presence of a TM and the corresponding antigen. Thus, the approach offers an inherent switch to turn CAR T reactivity on and off by simply applying or withdrawing TM supply and allows a timely restricted antitumor response. Upon stopping TM infusion, normal hematopoiesis should recover. Moreover, it adds additional flexibility to CAR T-cell therapy by simply exchanging TMs or applying multispecific TMs. Targeting both antigens with a single bispecific TM enhances the antileukemia efficacy as demonstrated by the very low concentrations of the dual-specific anti-CD123-CD33 TM required to induce lysis of AML samples. Moreover, targeting multiple antigens simultaneously or subsequently counteracts the risk for development of tumor escape variants under treatment, as already observed in recent clinical trials.^7, 8^

Multiple studies indicate that incorporation of additional costimulatory signals are required for CAR T cells to avoid anergy, to be fully activated and to sustain their expansion.^39, 40, 41^ Therefore, we constructed the UniCAR as a second-generation CAR with CD28 intracellular domain as part of the CAR signaling chain. CD28 signaling enables engineered T cells to synthesize and secrete IL-2 upon CAR engagement (Figure 2e). IL-2 in turn stimulates proliferation in an auto- and paracrine manner. Moreover, CD28 signaling prevents activated T cells from undergoing activation-induced cell death,^42^ enhancing persistence of the modified T cells.^43^ Inert UniCAR T cells might persist even lifelong in patients as already observed for conventional CAR T cells^44^ and could be reactivated as a universal weapon against malignant or infectious diseases at any time by simply infusing a disease-adjusted TM.

As shown by our *in vitro* and *in vivo* results, UniCAR engineered T cells remain inactive in the absence of TMs and their corresponding surface-bound antigen. Keeping in mind that the target epitope of the UniCAR receptor is highly conserved between humans and mice,^45^ the *in vivo* models are of high predictive value for the clinical situation regarding any unwanted side effects mediated by UniCAR T cells alone. It is an important finding that UniCAR modified human T cells do not cause any signs of autoimmunity neither in the absence of TMs nor under treatment conditions in our mouse model. The La protein is a known auto-antigen in Sjögren's syndrome and systemic lupus erythematosus and autoantibodies against La epitopes are common in patients, but might even preexist in healthy populations. One could argue that preexisting or under UniCAR T-cell therapy newly developed autoantibodies against the epitope used in the UniCAR system could interfere with the therapy by blocking the epitope on TMs and/or triggering autoimmunity. However, large screens of systemic lupus erythematosus and Sjögren's syndrome patient's sera for autoantibodies against La epitopes did not identify autoantibodies reactive with the La/SS-B-derived peptide domain used in the UniCAR system (MP Bachmann, unpublished, and see, for example, Yiannaki *et al.*^46^). Therefore, the induction of autoimmune reactions by UniCAR therapy appears rather unlikely.

Taken together, the modular composition of the UniCAR platform maintains the high antitumor potential of CAR engrafted T cells while introducing real control mechanisms and unparalleled target flexibility. Major advantages of this technology include (1) short development time for TMs directed against new targets, (2) possibility of rapidly interrupting an ongoing therapy by stopping the TM supply and (3) flexibility in case of tumor escape variants. These features will allow a more sophisticated application of CAR technology and a reduction of adverse events in the clinical setting.
