# Supplementary figures and images for: Switching CAR T cells on and off: a novel modular platform for retargeting of T cells to AML blasts
Source: Blood Cancer J. 2016 Aug 12;6(8):e458–. doi: 10.1038/bcj.2016.61 (PMC5022178; doi:10.1038/bcj.2016.61)

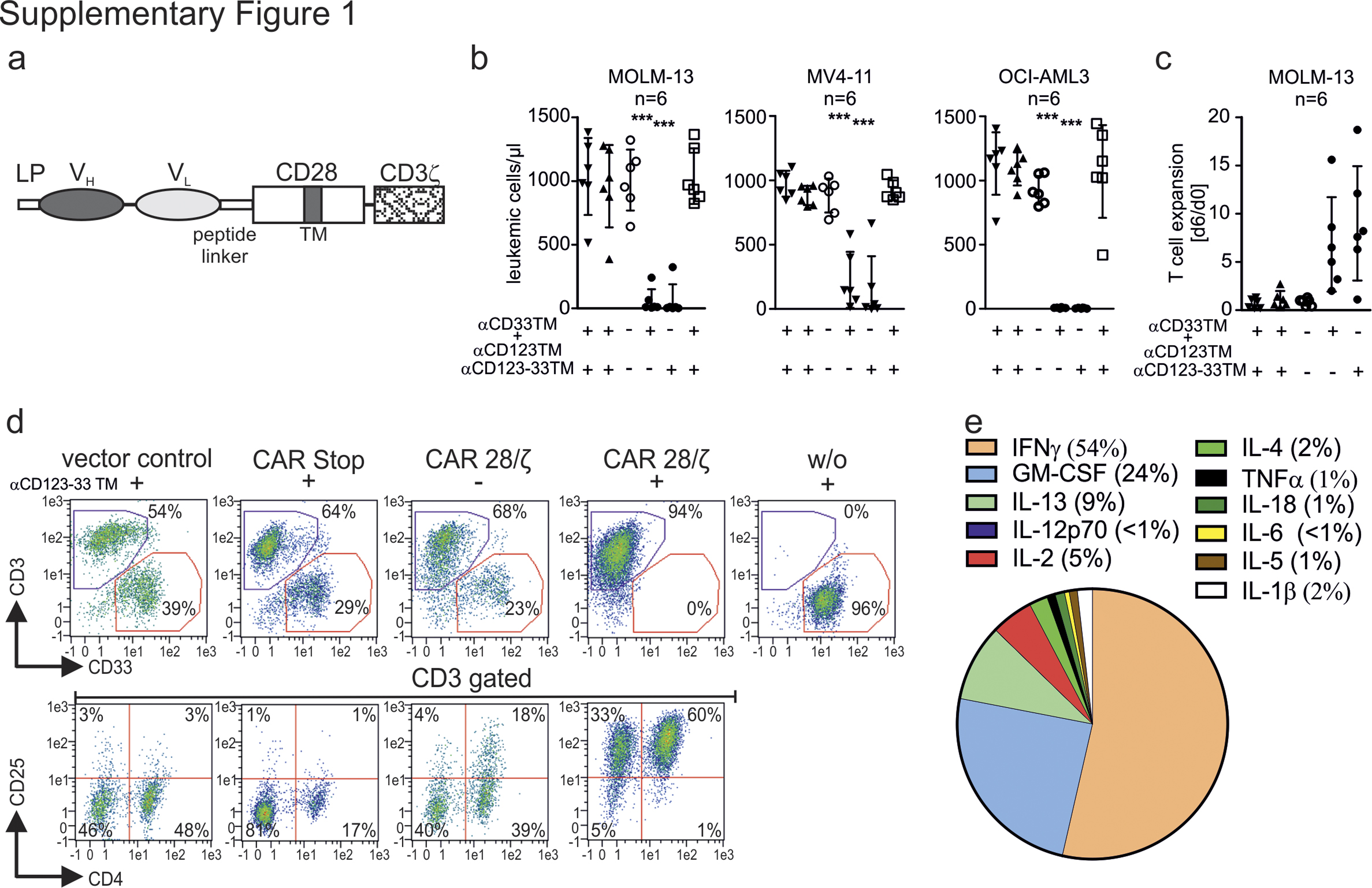

Supplement: Supplementary Figure 1 [file bcj201661x1.tif]
