# Supplementary material for: Switching CAR T cells on and off: a novel modular platform for retargeting of T cells to AML blasts
Source: Blood Cancer J. 2016 Aug 12;6(8):e458–. doi: 10.1038/bcj.2016.61 (PMC5022178; doi:10.1038/bcj.2016.61)
Supplement: Supplementary Figure 1 Legend [file bcj201661x2.doc]

**Supplementary Figure 1:** **UniCAR T cells can be redirected against CD33 and CD123 simultaneously**. (**a**) Scheme of UniCAR. The scFv is fused to human CD28 (aa 22-220) via a short peptide linker recognized by the anti-La 7B6 mAb. The CD28 stop codon is deleted and instead replaced by a glycine-serine linker (4xG_4_S_1_) and fused to the signaling subunit of the human CD3ζ chain from aa 55-220. The B7-binding motif within the extracellular domain of CD28 (MYPP→MAPPA) and an internalization motif at the N-terminal half of the signaling domain of CD28 (LL→GG) are both mutated and non-functional. (**b, c**) Human T cells were engineered to express functional UniCARs (open and closed circles), UniCARs lacking any signaling domain (head up triangle) or expressing only EGFP (head down triangle). Engineered T cells were incubated with 2*10^4^ target cells in the presence (+) or absence (-) of total amount of 100 pMol TMs specific for CD33 (αCD33 TM), CD123 (αCD123 TM) or bispecific CD123/CD33 (αCD123-CD33 TM) at an effector to target (e:t) ratio of 1:5 for 144h. (**b**) Number of living target cells was determined by flow cytometry and compared to control samples with target cells but without the addition of T cells (open squares). (**c**) T cell expansion was calculated as the ratio of T cells present in the samples after 144h (d6) to the number of cells seeded at the start of the experiment (d0). Results from experiments with six donors are shown, mean and s.d. are indicated. (**d**) Allogenic UniCAR modified T cells were incubated with 5*10^4^ leukemic cells from an AML patient in the presence (+) or absence (-) of anti-CD123-CD33 TM at 5 nMol and an e:t ratio of 1:1. After 120h of incubation, cells were harvested and stained with anti-CD3, anti-CD4, anti-CD8, anti-CD25 and anti-CD33 fluorochrom-labeled monoclonal antibodies. (**e**) Cytokine composition released from TM-redirected UniCAR engineered T cells upon incubation with allogenic leukemic cells from AML patients after 48h at an e:t ratio of 1:1. Statistical analysis for (**b**) was performed using non-parametric one-way ANOVA (Kruskal-Wallis test) and post-hoc Dunn’s Multiple Comparison test. Results are indicated for UniCAR modified T cells plus TMs versus other samples (*p < 0.05, **p < 0.01, ***p < 0.001).
